# Supplementary material for: CASPI: collaborative photon processing for active single-photon imaging
Source: Nat Commun. 2023 May 31;14:3158. doi: 10.1038/s41467-023-38893-9 (PMC10232480; doi:10.1038/s41467-023-38893-9)
Supplement: Supplementary file 1 — Supplementary Information [file 41467_2023_38893_MOESM1_ESM.pdf]

Supplementary Information for

CASPI: Collaborative Photon Processing for Active Single-Photon Imaging

Jongho Lee[1*], Atul Ingle[2], Jenu V. Chacko[3,4], Kevin W. Eliceiri[3,4,5,6,7,8] & Mohit Gupta[1,8]

[1] *Department of Computer Sciences, University of Wisconsin-Madison, Madison, WI, United States*

[2] *Department of Computer Science, Portland State University, Portland, OR, United States*

[3] *Laboratory for Optical and Computational Instrumentation, University of Wisconsin-Madison, Madison, WI, United States*

[4] *Center for Quantitative Cell Imaging, University of Wisconsin-Madison, Madison, WI, United States*

[5] *Morgridge Institute for Research, Madison, WI, United States*

[6] *Department of Biomedical Engineering, University of Wisconsin-Madison, Madison, WI, United States*

[7] *Department of Medical Physics, University of Wisconsin-Madison, Madison, WI, United States*

[8] *McPherson Eye Research Institute, Madison, WI, United States*

*Corresponding author: jlee567@wisc.edu

Contents

1 Supplementary Derivation **3**

1.1 Derivation of Noise Threshold (Eq. 9) 3

2 Supplementary Discussion **4**

2.1 Comparisons with BM4D and V-BM4D 4

3 Supplementary Tables **5**

4 Supplementary Results **7**

4.1 Filtering after Estimation vs. Estimation after Flux Recovery 7

4.2 Intensity Estimation Comparisons 8

4.3 Depth Estimation when High-quality Intensity is Available 10

4.4 LiDAR Simulations at Different Spatial Resolutions 12

4.5 Stress Test in FLIM 13

4.6 Lifetime Estimation Accuracy 15

4.7 Lifetime Estimation with Spatial Sequence of Photon Transient Cubes 16

1 Supplementary Derivation

1.1 Derivation of Noise Threshold (Eq. 9)

Let $\mathcal{R}$ and $\mathcal{I}$ be the real and imaginary parts of the Fourier coefficients inside the pure noise band of the 1D photon transient, 3D photon transient cubelet, or 4D photon transient set. According to the central limit theorem, $\mathcal{R}$ and $\mathcal{I}$ follow a Gaussian distribution with zero mean and standard deviation σ_N: $\mathcal{R} \sim \mathcal{N}\left(0, \sigma_N\right)$ and $\mathcal{I} \sim \mathcal{N}\left(0, \sigma_N\right)$. It is known that if $\mathcal{R} \sim \mathcal{N}\left(0, 1\right)$ and $\mathcal{I} \sim \mathcal{N}\left(0, 1\right)$, the noise magnitude $\mathcal{M} = \sqrt{\mathcal{R}^2 + \mathcal{I}^2}$ follows the chi distribution with two degrees of freedom ($\mathcal{M} \sim \chi\left(2\right)$). Therefore,

$$\sqrt{\left(\frac{\mathcal{R}-0}{\sigma_N}\right)^2 + \left(\frac{\mathcal{I}-0}{\sigma_N}\right)^2} = \frac{1}{\sigma_N}\sqrt{\mathcal{R}^2 + \mathcal{I}^2} = \frac{\mathcal{M}}{\sigma_N} \sim \chi\left(2\right). \tag{S1}$$

For the chi distribution with two degrees of freedom, the mean and standard deviations are defined as:

$$\mathbb{E}\left[\frac{\mathcal{M}}{\sigma_N}\right] = \sqrt{2}\frac{\Gamma(3/2)}{\Gamma(1)} \tag{S2}$$

and

$$std\left[\frac{\mathcal{M}}{\sigma_N}\right] = \sqrt{2}\sqrt{1-\left(\frac{\Gamma(3/2)}{\Gamma(1)}\right)^2}, \tag{S3}$$

where $\mathbb{E}\left[\cdot\right]$ and $std\left[\cdot\right]$ are the mean and standard deviation operators, respectively. Therefore,

$$\mathbb{E}\left[\mathcal{M}\right] = \sigma_N\sqrt{2}\frac{\Gamma(3/2)}{\Gamma(1)} \tag{S4}$$

and

$$std\left[\mathcal{M}\right] = \sigma_N\sqrt{2}\sqrt{1-\left(\frac{\Gamma(3/2)}{\Gamma(1)}\right)^2}. \tag{S5}$$

We define the noise threshold δ_{noise} as $\mathbb{E}\left[\mathcal{M}\right] + 4std\left[\mathcal{M}\right]$ (statistical upper bound of the noise magnitude $\mathcal{M}$). Then

$$\frac{\delta_{\text{noise}}}{\mathbb{E}\left[\mathcal{M}\right]} = \frac{\mathbb{E}\left[\mathcal{M}\right] + 4std\left[\mathcal{M}\right]}{\mathbb{E}\left[\mathcal{M}\right]} = 1 + 4\sqrt{\left(\frac{\Gamma(1)}{\Gamma(3/2)}\right)^2 - 1} = 1 + 4\sqrt{\frac{4}{\pi} - 1}. \tag{S6}$$

$$\Rightarrow \delta_{\text{noise}} = \left(1 + 4\sqrt{\frac{4}{\pi} - 1}\right)\mathbb{E}\left[\mathcal{M}\right]. \tag{S7}$$

2 Supplementary Discussion

2.1 Comparisons with BM4D and V-BM4D

Reliable local noise statistics are required for BM4D and V-BM4D to produce optimal results. Although BM4D and V-BM4D feature an optional automatic local noise estimation procedure[1], it frequently fails for photon transient cubes because noise is estimated from arbitrary high-frequency components that are not matched with the photon transient cubes for active imaging. Hand-tuning of noise parameters is not feasible for many active imaging scenarios, where the local SNR changes dynamically due to spatially and temporally varying illumination conditions. In contrast, CASPI automatically adapts to severely noisy operating scenarios by estimating local noise accurately in the pure noise band without requiring any prior knowledge of noise statistics. Our method provides higher quality flux estimates and depth estimates than the state-of-the-art BM4D/V-BM4D approaches over various illumination conditions (Fig. 3c).

3 Supplementary Tables

$\mathrm{SBR_{total}}$: ratio between the total number of signal photon counts N_{sig} and background photon counts N_{bkg} received over the entire exposure time ($N_{\mathrm{cycle}} \times T$)

$\mathrm{SBR_{pulse}}$: ratio between the number of signal photon counts N_{sig} and the number of background photon counts n_{bkg} received during the laser pulse peak duration (e.g., FWHM for a Gaussian pulse)

N_{cycle}: total number of laser cycles

N_x: number of columns of the photon transient cube

N_y: number of rows of the photon transient cube

N_t: number of time bins

Δ_t: time bin size

T: laser cycle period ($\Delta_t \times N_t$)

N_c: number of the photon transient cubes

Scene	$\mathrm{SBR_{total}}$ = $N_{\mathrm{sig}}/N_{\mathrm{bkg}}$	$\mathrm{SBR_{pulse}}$ = $N_{\mathrm{sig}}/n_{\mathrm{bkg}}$	N_{cycle}	N_x	N_y	N_t	Δ_t	T	N_c
Art (Figs. 1c, 2, 10, 3a, 3c, S1, S2, S3, S4)	$2/50$	8.2	$1,000$	209	167	$1,024$	80 ps	82 ns	1
Laundry (Figs. 3a, 3c, S2, S3)	$0.2/10$	4.1	$1,000$	202	167	$1,024$	80 ps	82 ns	1
Bowling1 (Figs. 3a, S2, S3)	$10/2,000$	1.0	$1,000$	188	167	$1,024$	80 ps	82 ns	1
Road (Fig. 3a)	$3,000/3,000$	204.8	$1,000$	200	200	$1,024$	680 ps	696 ns	20
Kitchen (Fig. 5)	$10/0$	∞	$1,000$	320	240	$2,000$	33 ps	66 ns	1
Reindeer (Figs. S2, S3)	$100/100$	204.8	$1,000$	202	167	$1,024$	80 ps	82 ns	1
Moebius (Fig. S3)	$0.1/0$	∞	$1,000$	209	167	$1,024$	80 ps	82 ns	1

Table S1: Photon transient cube specification for LiDAR simulations.

Scene	$\text{SBR}_{\text{total}} = N_{\text{sig}}/N_{\text{bkg}}$	$\text{SBR}_{\text{pulse}} = N_{\text{sig}}/n_{\text{bkg}}$	N_{cycle}	N_x	N_y	N_t	Δ_t	T	N_c
Deer (Fig. 4a)	$2/320$	0.9	30	154	174	$8,192$	$8\,\text{ps}$	$66\,\text{ns}$	1
Face (Fig. 4b)	$24/0$	∞	90	116	204	$8,192$	$8\,\text{ps}$	$66\,\text{ns}$	1
Face (Fig. 4b)	$2.4/0$	∞	9	116	204	$8,192$	$8\,\text{ps}$	$66\,\text{ns}$	1
Face (Fig. 4b)	$0.8/0$	∞	3	116	204	$8,192$	$8\,\text{ps}$	$66\,\text{ns}$	1

Table S2: Photon transient cube specification for LiDAR experiments.

Sample	$\text{SBR}_{\text{total}} = N_{\text{sig}}/N_{\text{bkg}}$	N_{cycle}	N_x	N_y	N_t	Δ_t	T	N_c
Fig. 7	$200/0$	$1,000$	256	256	256	$48\,\text{ps}$	$12\,\text{ns}$	1

Table S3: Photon transient cube specification for FLIM simulations.

Sample	$\text{SBR}_{\text{total}} = N_{\text{sig}}/N_{\text{bkg}}$	N_{cycle}	N_x	N_y	N_t	Δ_t	T	N_c
Figs. 1d, 6b	$30/0$	$20,000$	256	256	256	$48\,\text{ps}$	$12\,\text{ns}$	497
Fig. 6a	$10/0$	800	256	256	256	$48\,\text{ps}$	$12\,\text{ns}$	1
Fig. 6c	$40/0$	$1,600$	256	256	256	$48\,\text{ps}$	$12\,\text{ns}$	261
Fig. S5	$10/0$<br>$20/0$<br>$40/0$<br>$80/0$<br>$2,500/0$	800<br>$1,600$<br>$3,200$<br>$6,400$<br>$204,800$	256	256	256	$48\,\text{ps}$	$12\,\text{ns}$	1
Fig. S6	$13/0$	$12,000$	256	256	256	$48\,\text{ps}$	$12\,\text{ns}$	60
Fig. S7	$9/0$	$18,000$	256	256	256	$48\,\text{ps}$	$12\,\text{ns}$	16

Table S4: Photon transient cube specification for FLIM experiments.

4 Supplementary Results

4.1 Filtering after Estimation vs. Estimation after Flux Recovery

We compare between conventional image filtering after depth estimation and depth estimation after CASPI. Given the noisy photon transient cube under the flux regime of $\text{SBR}_{\text{total}} = 2/50$ ($\text{SBR}_{\text{pulse}} = 8.3$), we obtain the noisy depth map by traditional matched filtering as shown in Fig. S1. Since the noise of the estimated depth map in this challenging lighting condition does not follow commonly assumed noise models, the conventional image filtering such as median filtering[2] (obtained the best result when mask size is 3×3) and BM3D[3] (obtained the best result when the input noise variance = 1) cannot recover reliable depth estimates as shown in Fig. S1. However, after the latent photon fluxes are recovered by CASPI, we can generate the high-quality depth map even with traditional matched filtering as shown in Fig. S1. This suggests that denoising early in the photon processing chain is key to achieving optimal results.

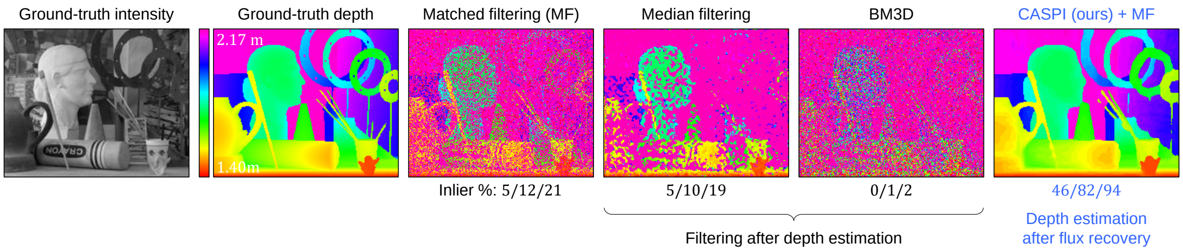

Fig. S1: Filtering after estimation vs. estimation after filtering. Depth estimation followed by conventional image filtering fails to get reliable depth estimates under challenging lighting conditions. After recovering the latent photon fluxes using CASPI, we can obtain reliable depth estimates even with a simple matched filtering.

4.2 Intensity Estimation Comparisons

Figure S2 shows intensity estimation comparisons between different approaches under various lighting conditions with the Middlebury dataset[4]. After recovering photon fluxes with CASPI, we obtain the intensity estimates by simply summing over the time dimension of the recovered photon fluxes. We compare our results with photon counting (PC), and two statistical approaches (statistical I[5], statistical II[6]). Root-mean-square error (RMSE) is used for the objective performance measure. As shown in Fig. S2, the proposed approach provides higher quality scene intensity estimates than the compared approaches over a wide range of flux regimes. For example, CASPI preserves intensity details better than the compared approaches in high signal-to-background ratio (SBR) regime ($\text{SBR}_{\text{total}} = 100/100$) while suppressing the noise more effectively than the other approaches in sub-photon regime ($\text{SBR}_{\text{total}} = 0.2/10$). Furthermore, in high background flux regime ($\text{SBR}_{\text{total}} = 10/2,000$), the saturated foreground and the excessive high contrast region of the bowling ball in the photon counting result are recovered well with our approach.

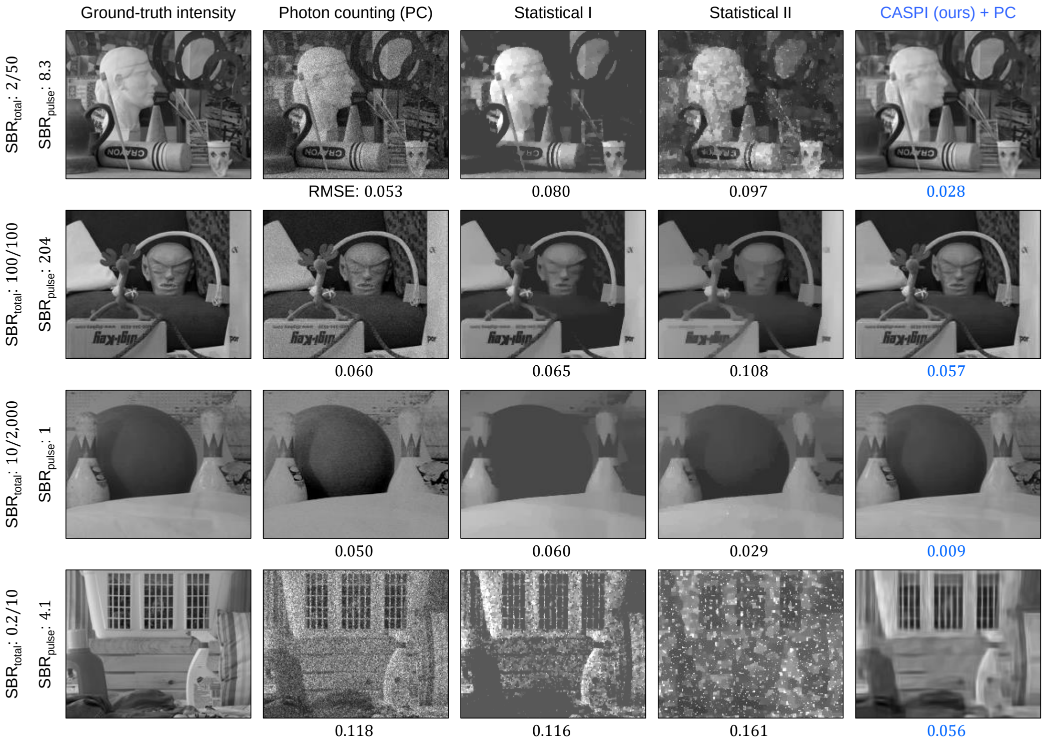

Fig. S2: Scene intensity estimation comparisons. CASPI enables high-quality scene intensity estimates by simply summing over the time dimension of the recovered photon fluxes. Compared to other approaches, our method preserves intensity details better in high signal-to-background ratio (SBR) regime ($\text{SBR}_{\text{total}} = 100/100$) while suppressing the noise more effectively in sub-photon regime ($\text{SBR}_{\text{total}} = 0.2/10$). In addition, the saturation and the excessive high contrast in the photon counting result are also reduced by our approach when operating in a high background flux regime ($\text{SBR}_{\text{total}} = 10/2,000$). Root-mean-square error (RMSE) is labeled below each image as the objective performance measure.

4.3 Depth Estimation when High-quality Intensity is Available

Although CASPI provides reliable intensity estimates as output, we can also use high-quality intensity images as input to get better depth estimates. Additional high-quality intensity information is beneficial for similar cubelet finding and guided photon processing in CASPI. Figure S3 shows depth estimation results with and without the additional intensity images as input over a wide range of lighting conditions. For comparisons, we show the results by the learning-based approach[7]. They provide two types of trained models with and without the intensity images as input. In general, we can obtain better depth estimates with the intensity images for both approaches as shown in Fig. S3. Note that our method without the intensity images provides better depth estimates than the learning-based approach[7] with the intensity images under all test lighting conditions.

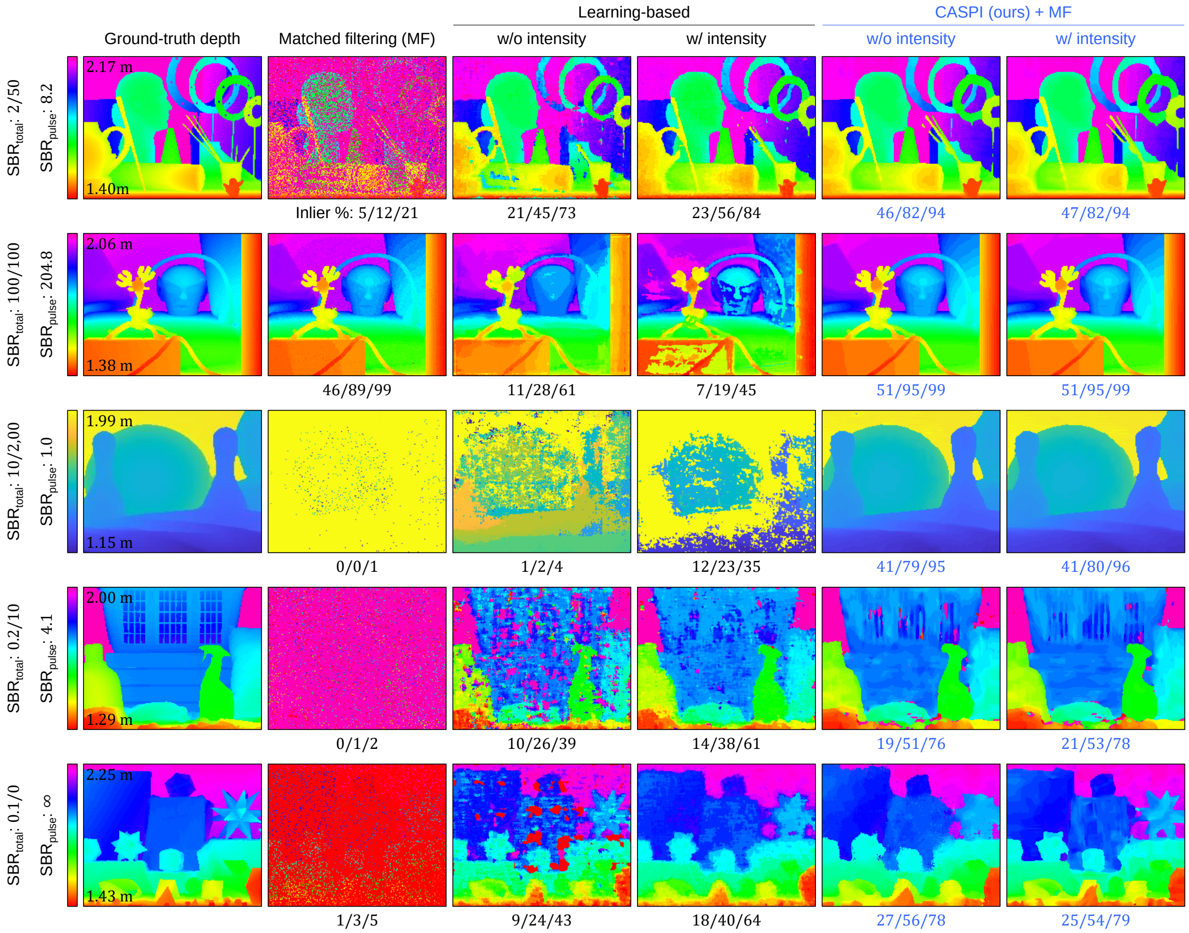

Fig. S3: Depth estimation with high-quality intensity input. If high-quality intensity images are available as side input, we can obtain better depth estimates with CASPI. We compare our approach with the learning-based approach[7] which provides two types of trained models with and without the intensity images. Although intensity information improves the performance of both approaches, our method without the intensity images provides better depth estimates than the learning-based approach with the intensity images under all test lighting conditions.

4.4 LiDAR Simulations at Different Spatial Resolutions

Depth and intensity estimation performance depends on the spatial resolution of the photon transient cube. We can get better estimates when the spatial resolution increases. Figure S4a shows depth estimates by traditional matched filtering, statistical approach II[6], learning-based approach[7], and our approach followed by matched filtering. Figure S4b shows intensity estimates by photon counting, statistical approach I[5], statistical approach II[6], and our method followed by photon counting (summing over the time dimension of the photon transient cube). Test flux regime is $\text{SBR}_{\text{total}} = 2/50$. Upper and lower rows show the results when the spatial resolution is 167×209 and 555×695, respectively. Most approaches show improved depth and intensity estimates when the spatial resolution increases. CASPI shows the best performance compared to other approaches at any spatial resolution.

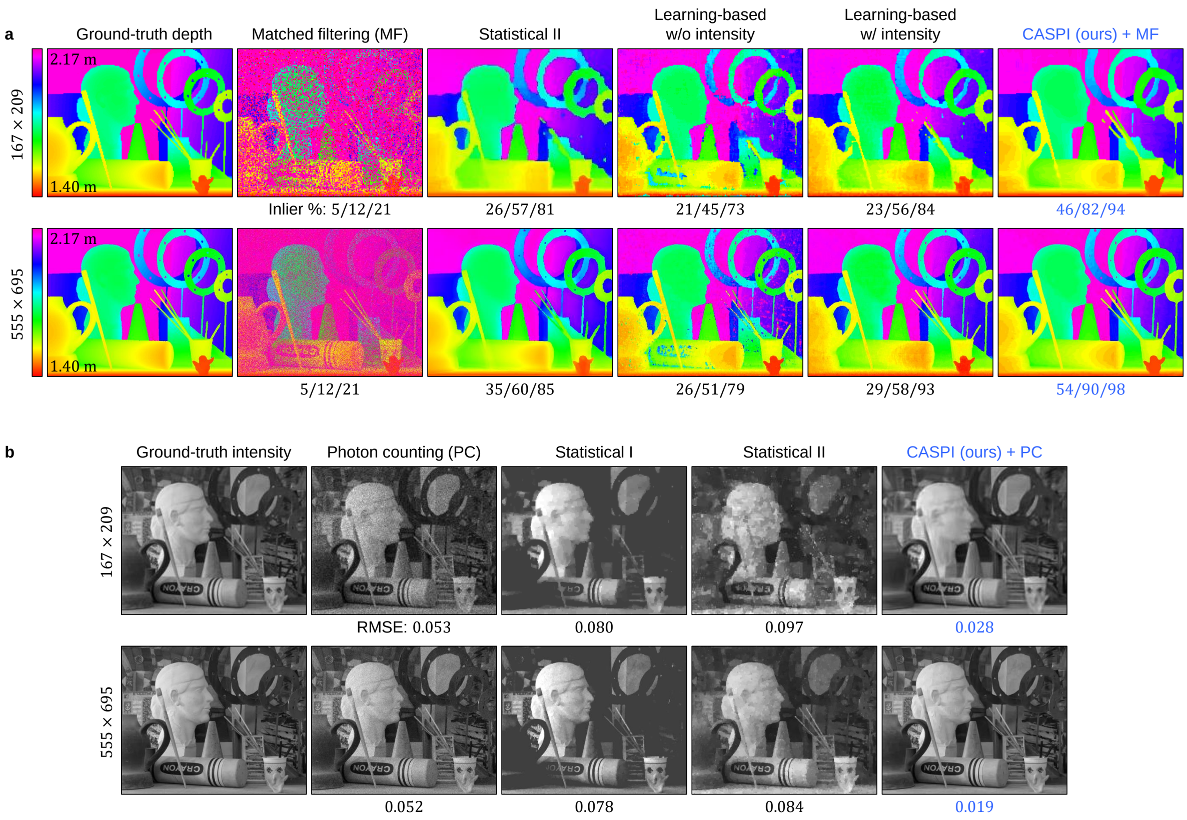

Fig. S4: Depth estimation at different spatial resolutions. a, **b**. (**a**) Depth and (**b**) intensity estimation performance increases with the spatial resolution of photon data. CASPI shows the best performance compared to other approaches in terms of both depth estimation and intensity estimation at any spatial resolution.

4.5 Stress Test in FLIM

In order to study controlled photon-starved conditions, we imaged live cells expressing mCherry-H2B fluorescent tags (see Methods). With a photon count rate of 100 photons/sec/frame, multiple data sets with different photon counts were obtained by accumulating for different periods of time. The average photon counts per pixel of these photon transient cubes are about 10, 20, 40, 80, and $2,500$ as shown in Fig. S5. The photon transient cube with $2,500$ photons/pixel is used as ground-truth data. Each cube is processed in three different ways for comparisons: no processing (raw data), 7×7 spatial binning (the binning size is comparable to the spatial size of the photon cubelet of our approach), and CASPI. The lifetimes are fitted using MLE in the SPCImage[8] (Becker Hickl GmbH, Berlin). Figure S5a shows the lifetime images estimated from these three processed photon cubes with different photon counts. Our approach enables reliable lifetime images by recovering true photon fluxes even with small photon counts (as low as 10 photons/pixel) as shown in Fig. S5b.

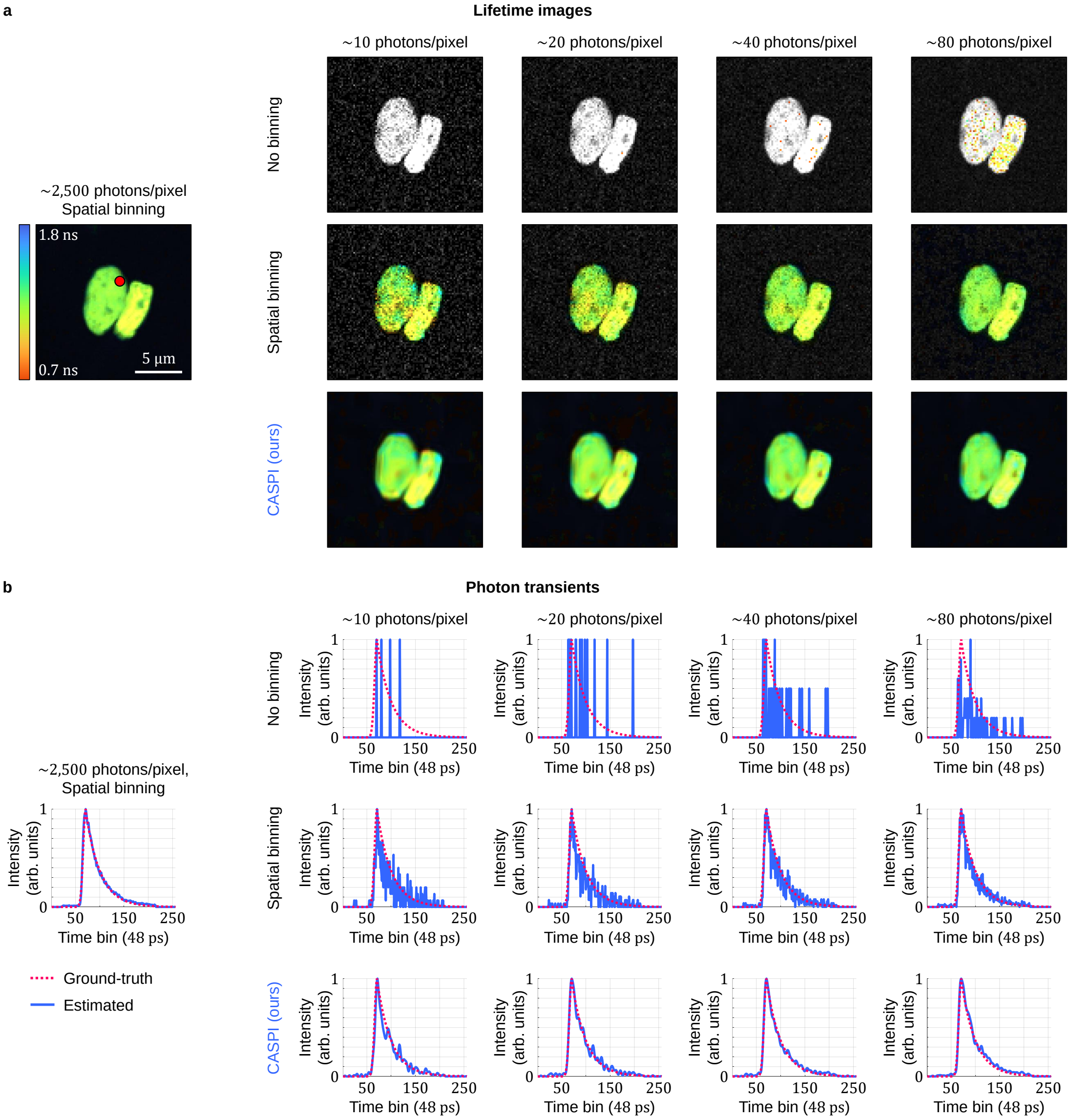

Fig. S5: Stress test. a, b. CASPI provides (**a**) higher lifetime estimation accuracy than spatial binning even with as few as 10 photons/pixel by successfully recovering (**b**) the temporal fluorescence emission.

4.6 Lifetime Estimation Accuracy

We also test the accuracy of lifetime estimation with CASPI in low SNR scenarios. We captured a time-lapse sequence of the photon transient cubes of mCherry-H2B tags in HeLa cells (see Methods). MCherry has a known fluorescence lifetime of $1.4\,\mathrm{ns}$[9]. We process the photon measurements with 7×7 spatial binning and CASPI, and we compare the lifetime estimation results. Figure S6 shows the comparisons in terms of the estimated lifetime images and the estimated lifetime histograms. With CASPI, we can get lifetime estimates for more pixels, and the estimated lifetimes are better clustered around $1.4\,\mathrm{ns}$, which is the ground-truth value.

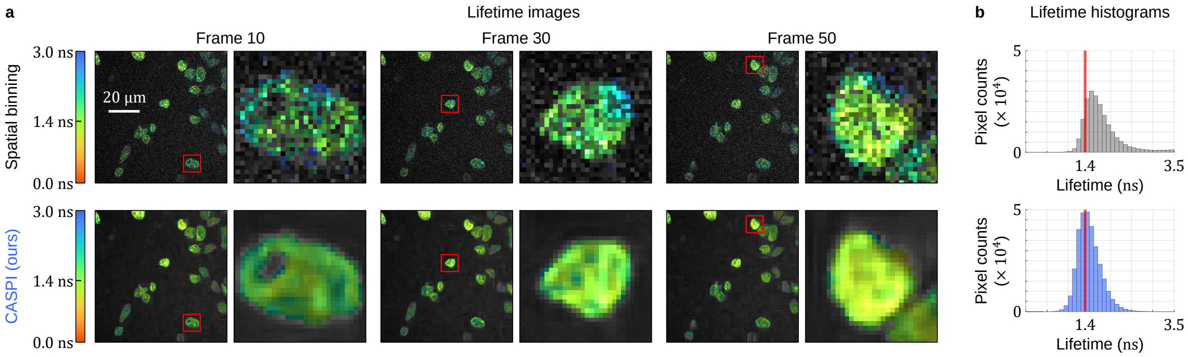

Fig. S6: Lifetime estimation accuracy. a, b. The fluorescence emission measurements of the MCherry stained cell nuclei which has a known lifetime of $1.4\,\mathrm{ns}$ are captured in low SNR illumination condition. The photon measurements are processed with 7×7 spatial binning and CASPI, and the lifetime estimates are compared in terms of (**a**) lifetime images and (**b**) lifetime histograms. With CASPI, we can get more accurate lifetimes for more number of pixels in the image.

4.7 Lifetime Estimation with Spatial Sequence of Photon Transient Cubes

CASPI can be applied not only to the temporal sequence of the photon transient cubes but also to the spatial sequence. We obtained the 3D/Z-stack data of the plated cellular pellets using their intrinsic autofluorescence (see Methods). Non-local correlations between the cubes at different spatial positions can be exploited to recover the latent photon fluxes by our approach. We process the photon measurements with 7×7 spatial binning and CASPI, and we compare the lifetime estimation results. Figure S7 shows the lifetime estimation comparisons with the Z-stack data.

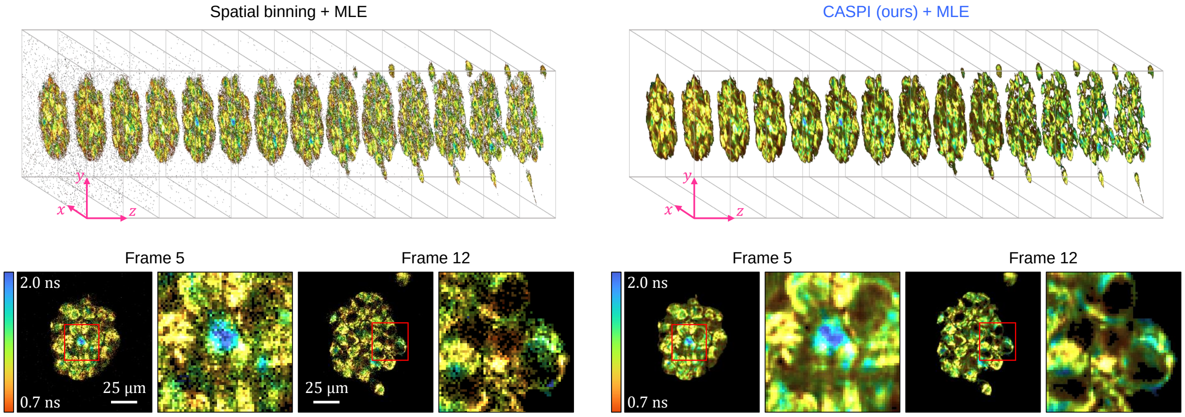

Fig. S7: FLIM results with spatial sequence of photon data. CASPI can be applied to not only the temporal sequence of the photon transient cubes but also the spatial sequence such as the Z-stack, where non-local correlations can also be exploited to recover photon fluxes. The improved morphological details without noise help to make 3D FLIM acquisition faster and avoid laser-induced photobleaching.

Supplementary References

1. Maggioni, M. & Foi, A. Nonlocal transform-domain denoising of volumetric data with groupwise adaptive variance estimation. In *Computational Imaging X*, vol. 8296, 133–140 (SPIE, 2012).

2. Lim, J. S. Two-dimensional signal and image processing. *Englewood Cliffs* (1990).

3. Dabov, K., Foi, A., Katkovnik, V. & Egiazarian, K. Image denoising by sparse 3-d transform-domain collaborative filtering. *IEEE Transactions on image processing* **16**, 2080–2095 (2007).

4. Scharstein, D. & Pal, C. Learning conditional random fields for stereo. In *2007 IEEE Conference on Computer Vision and Pattern Recognition*, 1–8 (IEEE, 2007).

5. Shin, D., Kirmani, A., Goyal, V. K. & Shapiro, J. H. Photon-efficient computational 3-d and reflectivity imaging with single-photon detectors. *IEEE Transactions on Computational Imaging* **1**, 112–125 (2015).

6. Rapp, J. & Goyal, V. K. A few photons among many: Unmixing signal and noise for photon-efficient active imaging. *IEEE Transactions on Computational Imaging* **3**, 445–459 (2017).

7. Lindell, D. B., O'Toole, M. & Wetzstein, G. Single-photon 3d imaging with deep sensor fusion. *ACM Trans. Graph.* **37**, 113–1 (2018).

8. Bergmann, A. Spcimage: data analysis software for fluorescence lifetime imaging microscopy. *Becker & Hickl GmbH, available on www. becker-hickl. com* (2003).

9. Merzlyak, E. M. *et al.* Bright monomeric red fluorescent protein with an extended fluorescence lifetime. *Nature methods* **4**, 555–557 (2007).
