# Supplementary material for: CASPI: collaborative photon processing for active single-photon imaging
Source: Nat Commun. 2023 May 31;14:3158. doi: 10.1038/s41467-023-38893-9 (PMC10232480; doi:10.1038/s41467-023-38893-9)
Supplement: Supplementary file 3 — Description of Additional Supplementary Files [file 41467_2023_38893_MOESM3_ESM.docx]

**Description of Additional Supplementary Files**

**Supplementary Movie 1:**

This supplementary video provides motivation, implications, and results of our paper 'CASPI: Collaborative Photon Processing for Active Single-Photon Imaging'.
